# Respiratory Tract Explant Infection Dynamics of Influenza A Virus in California Sea Lions, Northern Elephant Seals, and Rhesus Macaques

**DOI:** 10.1128/JVI.00403-21

**Published:** 2021-07-26

**Authors:** Hongwei Liu, Magdalena Plancarte, Erin E. Ball, Christopher M. Weiss, Omar Gonzales-Viera, Karen Holcomb, Zhong-Min Ma, A. Mark Allen, J. Rachel Reader, Pádraig J. Duignan, Barbie Halaska, Zenab Khan, Divya Kriti, Jayeeta Dutta, Harm van Bakel, Kenneth Jackson, Patricia A. Pesavento, Walter M. Boyce, Lark L. Coffey

**Affiliations:** a University of California, Davis, School of Veterinary Medicine, Department of Pathology, Microbiology and Immunology, Davis, California, USA; b University of California, Davis, California National Primate Research Center, Davis, California, USA; c The Marine Mammal Center, Sausalito, California, USA; d Icahn School of Medicine at Mount Sinai, New York, New York, USA; Cornell University

**Keywords:** explant, infection dynamics, influenza A virus, kinetics, marine mammal, respiratory viruses, rhesus macaque, tropism

## Abstract

To understand susceptibility of wild California sea lions and Northern elephant seals to influenza A virus (IAV), we developed an *ex vivo* respiratory explant model and used it to compare infection kinetics for multiple IAV subtypes. We first established the approach using explants from colonized rhesus macaques, a model for human IAV. Trachea, bronchi, and lungs from 11 California sea lions, 2 Northern elephant seals, and 10 rhesus macaques were inoculated within 24 h postmortem with 6 strains representing 4 IAV subtypes. Explants from the 3 species showed similar IAV infection kinetics, with peak viral titers 48 to 72 h post-inoculation that increased by 2 to 4 log_10_ PFU/explant relative to the inoculum. Immunohistochemistry localized IAV infection to apical epithelial cells. These results demonstrate that respiratory tissue explants from wild marine mammals support IAV infection. In the absence of the ability to perform experimental infections of marine mammals, this *ex vivo* culture of respiratory tissues mirrors the *in vivo* environment and serves as a tool to study IAV susceptibility, host range, and tissue tropism.

**IMPORTANCE** Although influenza A virus can infect marine mammals, a dearth of marine mammal cell lines and ethical and logistical challenges prohibiting experimental infections of living marine mammals mean that little is known about IAV infection kinetics in these species. We circumvented these limitations by adapting a respiratory tract explant model first to establish the approach with rhesus macaques and then for use with explants from wild marine mammals euthanized for nonrespiratory medical conditions. We observed that multiple strains representing 4 IAV subtypes infected trachea, bronchi, and lungs of macaques and marine mammals with variable peak titers and kinetics. This *ex vivo* model can define infection dynamics for IAV in marine mammals. Further, use of explants from animals euthanized for other reasons reduces use of animals in research.

## INTRODUCTION

Influenza A viruses (IAV) are important etiologies of respiratory disease in humans and especially affect the elderly, infants, and people with immunodeficiencies and chronic respiratory disease. Dwarfed in 2020 by SARS-CoV-2, IAV are a significant cause of morbidity annually, producing about 500,000 deaths worldwide each year (1). IAV possess a wide host range that includes birds, horses, pigs, and humans (2, 3). Marine mammals can also be infected, sometimes with strains including H1N1 from human pandemics (4) or avian H3N8 (5). The viral genetic and host factors that affect cross-species transmission by IAV, especially from birds to mammals, including humans, have been extensively studied (5–9). However, mechanisms of interspecies IAV transmission from avian or human to other mammalian species, including marine mammals, are less well understood (6).

IAV was first identified in North American marine mammals in 1979, when an H7N7 epizootic killed 500 harbor seals and caused hemorrhagic pneumonia in others (10–12). Since then, IAV infection and sometimes disease have been identified in several marine mammal species, including mass mortalities in harbor seals near Cape Cod, MA, USA, attributed to H7N7, H4N5, or H4N6, and detection of antibody to multiple H and N subtypes in several seal species (13–20). On the West Coast, surveillance from 2009 to 2015 (4, 21, 22) in multiple marine mammal species from California shows variable IAV exposure. Seroprevalence in sea otters and Northern elephant seals is higher than in sympatric harbor seals and California sea lions, and sea otters are exposed to avian- and human-origin IAV, including pandemic H1N1 (4, 21). Despite frequent antibody detection, isolation of IAV from marine mammals has been limited. In California, only pandemic H1N1 was isolated from 2 Northern elephant seals in 2010 (4). Together, these surveillance data suggest that marine mammals can be infected with some of the same IAV subtypes that cause human epidemics. However, in the absence of capabilities to perform *in vivo* studies in marine mammals, infection dynamics and subtype-specific susceptibility remain unknown. Therefore, there is a need to develop approaches to study the infection biology of IAV in these species.

Given that the respiratory tract is the initial site of IAV infection, defining infection dynamics and cellular tropism in respiratory tissues is important for assessing species susceptibility. However, *in vivo* systems for studying susceptibility of marine mammals to IAV are not available. As an alternative, *ex vivo* explants from respiratory tract tissues can mimic the physiological microenvironment of a respiratory tract. *Ex vivo* systems in human and animals (excluding marine mammals) have been successfully developed and used to study host innate responses, infection dynamics, viral genetic determinants of infection, antiviral drug treatments, and pathogenesis of human and animal IAV (23–27) and other respiratory viruses (28). The goal of this study, therefore, was to expand these existing models for use in marine mammals to study susceptibility and infection dynamics of IAV strains of mammalian and avian origin to assess the potential for interspecies transmission and to provide a new approach for studying the biology and pathogenesis of IAV in marine mammals. Given that marine mammal tissues are only opportunistically available from wild animals treated at The California Marine Mammal Center in Sausalito, CA, USA, we first established the *ex vivo* explant system using rhesus macaques that are more regularly available from the California National Primate Research Center, Davis, CA, USA. Rhesus macaques represent a valuable model for understanding IAV infection dynamics in the human respiratory tract due to similar structure, physiology, and mucosal immunity. Additionally, IAV infection dynamics have not been studied in rhesus macaques, although they are a model for human IAV infection (29) and are used for testing vaccine candidates (30, 31).

We used trachea, bronchi, and lung explants from rhesus macaques to compare the susceptibility, infection kinetics, and tissue tropism of 6 strains of IAV from 4 subtypes to validate the utility of this system. After establishing the explant approach with IAV in rhesus macaques, we used it to study IAV infection dynamics and tropism in California sea lions and Northern elephant seals. We observed that both rhesus macaques and marine mammals are susceptible to all 6 IAV strains and that rhesus macaque and California sea lion respiratory tract explants exhibit temporal, tissue, and IAV subtype-dependent IAV infection kinetics.

## RESULTS

### Influenza A virus strains exhibit differential infection kinetics in immortalized cells.

**FIG 1:**
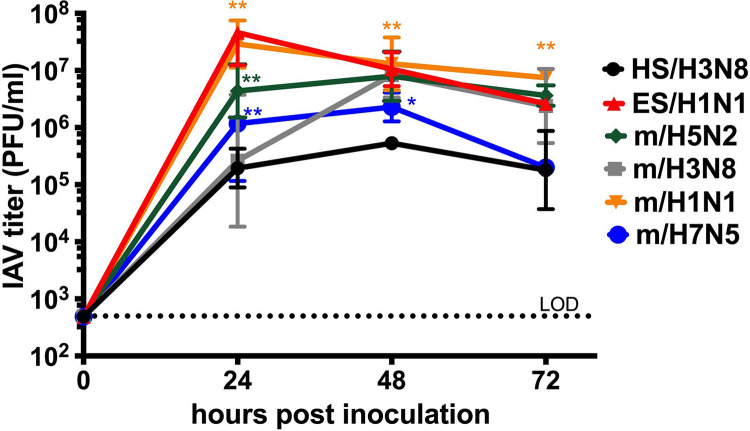
*In vitro* infection kinetics of 6 influenza A virus strains in Madin-Darby canine kidney cells. An MOI of 0.01 was used. The geometric mean virus titer from triplicate wells at each time point was plotted as PFU per milliliter ± geometric standard deviations. The dotted line represents the limit of detection (LOD) of 500 PFU/ml. Asterisks show comparisons of mean titers between HS/H3N8 and all other strains analyzed by two-way ANOVA of log-transformed values: *, *P < *0.001; **, *P < *0.0001. Strain names refer to the original source of the virus isolate followed by the hemagglutinin and neuraminidase subtype. HS, harbor seal. m, mallard. ES, elephant seal.

Given that cell infection kinetics for 4 of the strains had not previously been established, we first performed growth assays for each of the 6 IAV strains (Table 1) after inoculation into Madin-Darby canine kidney (MDCK) cells in triplicate at a multiplicity of infection (MOI) of 0.01 (Fig. 1). The H3N8 strain isolated from a harbor seal (HS/H3N8) exhibited the slowest and lowest growth kinetics, peaking at 10^5^ PFU/ml 48 h post-inoculattion (hpi). The H1N1 strains showed the fastest and highest growth kinetics, peaking at >10^7^ PFU/ml by 24 hpi. The viral infection kinetics of the other 3 strains isolated from mallard ducks were intermediate between HS/H3N8 and the 2 H1N1 strains. Titers for all strains were significantly higher than those for HS/H3N8 at one or more time points between 24 and 72 hpi (two-way analysis of variance [ANOVA]). Given these data show that *in vitro* infection kinetics in an immortalized cell line are different for the 6 IAV strains used in this study, we sought to determine whether the growth kinetics of these strains also differed in explant tissues from the 3 animal species.

**TABLE 1:** IAV strains used for explant infection experiments^*a*^

Virus ID	Strain name	Subtype	Passage no.	MDCK titer (PFU/ml)	GenBank accession no.	Reference
ES/H1N1	A/Elephant seal/California/1/2010	H1N1	p4	1.3 × 10^8^	JX865419–JX865426	4
HS/H3N8	A/harbor seal/New Hampshire/179629/2011	H3N8	p3	1.8 × 10^6^	KJ467564–KJ467571	5
m/H3N8	A/mallard/California/1475/2010	H3N8	p2	6 × 10^6^	CY120501–CY120508	NA
m/H1N1	A/mallard/California/3134/2010	H1N1	p2	4 × 10^7^	CY120611–CY120618	NA
m/H5N2	A/mallard/California/2396/2010	H5N2	p2	4 × 10^7^	CY120587–CY120594	NA
m/H7N5	A/mallard/California/1390/2010	H7N5	p2	1 × 10^7^	CY120555–CY120562	NA

a HS, harbor seal. m, mallard. ES, elephant seal. NA indicates strain was isolated at UC Davis and first described in this study.

### Influenza A viruses infect rhesus macaque respiratory tract explants.

**FIG 2:**
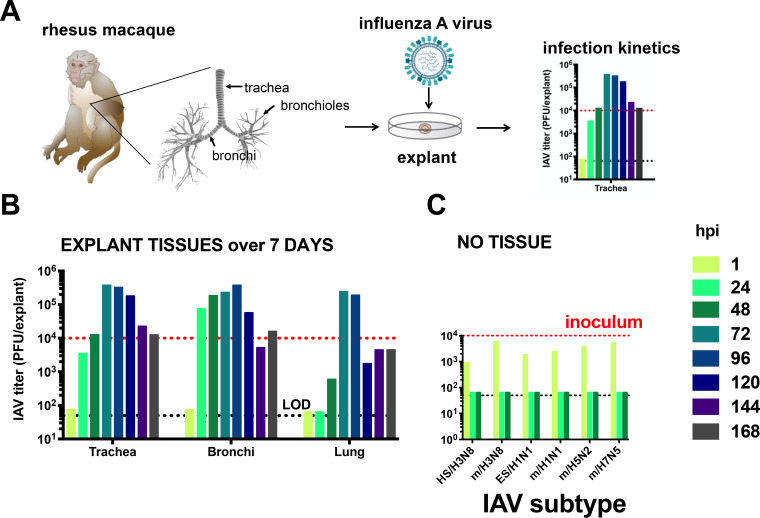
(A) Experimental design for *ex vivo* rhesus macaque respiratory tract explant inoculations with influenza A virus (IAV). (B) Seven-day infection kinetics of IAV HS/H3N8 in explants from an 11-year-old animal that was serologically IAV nonreactive at necropsy. (C) IAV titers in the absence of explants to evaluate stability of infectious virus from 0 to 48 hpi. Each bar shows the measurement from a single explant. The dotted black line shows the limit of detection, 50 PFU/explant. The red line shows the inoculum. Strain names refer to the original source of the virus isolate followed by the hemagglutinin and neuraminidase subtype. HS, harbor seal. m, mallard. ES, elephant seal.

Given that marine mammal tissues are only opportunistically available from wild animals treated at The California Marine Mammal Center in Sausalito, CA, USA, we first established the *ex vivo* explant system using rhesus macaques that are more regularly available from the California National Primate Research Center (CNPRC), Davis, CA, USA (Fig. 2A). The rhesus macaques were bred and then euthanized at CNPRC due to nonrespiratory medical conditions. Although tissues were used within 6 h postmortem, we microscopically visualized the movement of polystyrene beads by cilia placed atop tracheas from some macaques and verified viability at 24, 48, and 72 h, similar to Nunes et al. (23). To determine whether rhesus macaque explants support IAV infection and to define infection kinetics, we measured viral titers for 7 days (from 1 to 168 hpi) in explants from the first animal, an 11-year-old female macaque inoculated with HS/H3N8 (Fig. 2B) who had no detectable IAV antibody in sera at the time of necropsy. We defined infection as detection of a kinetic increase in infectious IAV titer above the inoculum, 10^4^ PFU. Titers increased over time in all 3 tissues and exceeded inocula from 48 to 168 hpi in trachea, from 24 to 120 hpi in bronchi, and from 72 to 96 hpi in the lung. Maximal titers of >10^5^ PFU/tissue were detected in all 3 tissue types. In contrast, when 10^4^ PFU of IAV strains were inoculated into medium in the absence of explants (Fig. 2C), no IAV was detected above the limit of detection (50 PFU/explant) at 24 or 48 hpi, which indicates IAV are not viable over time absent IAV-susceptible tissues. Together, these results demonstrate that rhesus macaque respiratory tract explants support productive infection by IAV.

### Influenza A virus exhibits strain-specific infection patterns in *ex vivo* respiratory tract tissues. (i) Rhesus macaques.

**FIG 3:**
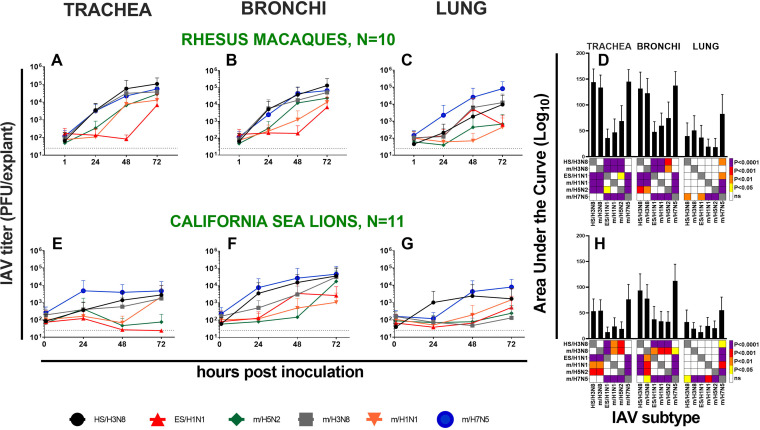
*Ex vivo* influenza A virus infection kinetics. (A to H) Mean kinetics in 10 rhesus macaque (A to C) and 11 California sea lions (E to G) and areas under the infection curve (AUC) (D and H) in *ex vivo* respiratory tract trachea, bronchi, and lung explants. Error bars show standard deviations. The dotted black line shows the limit of detection, 50 PFU/explant. Colors in squares in panels D and H show differences in mean AUC by strain analyzed using one-way ANOVA tests, where the darker the color, the smaller the *P* value. Strain names refer to the original source of the virus isolate followed by the hemagglutinin and neuraminidase subtype. HS, harbor seal. m, mallard. ES, elephant seal.

**FIG 4:**
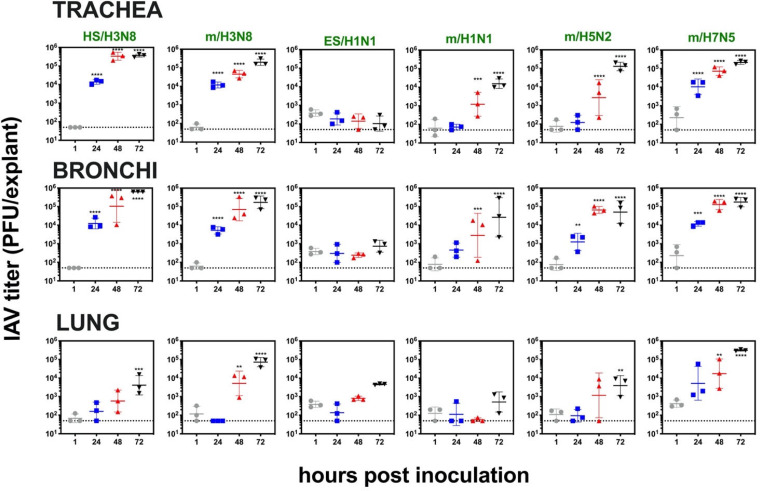
Influenza A virus infection kinetics from 1 to 72 hpi in respiratory tract explants from a 4-year-old male rhesus macaque (M15 in Table 2) inoculated with 1 × 10^4^ PFU of 6 viral strains. Viral titers are represented as the geometric mean and geometric standard deviation. Three explants at each time point were titrated independently, and the mean from the triplicates is represented by the middle horizontal line. Asterisks show *P* values (two-way ANOVA with Dunnett’s multiple comparisons) comparing titers at 24, 48, or 72 hpi to 1 hpi. **, *P* ≤ 0.005; ***, *P* < 0.001; ****, *P* < 0.0001. The dashed line indicates the limit of detection, 50 PFU/explant. Strain names refer to the original source of the virus isolate followed by the hemagglutinin and neuraminidase subtype. HS, harbor seal. m, mallard. ES, elephant seal.

**FIG 5:**
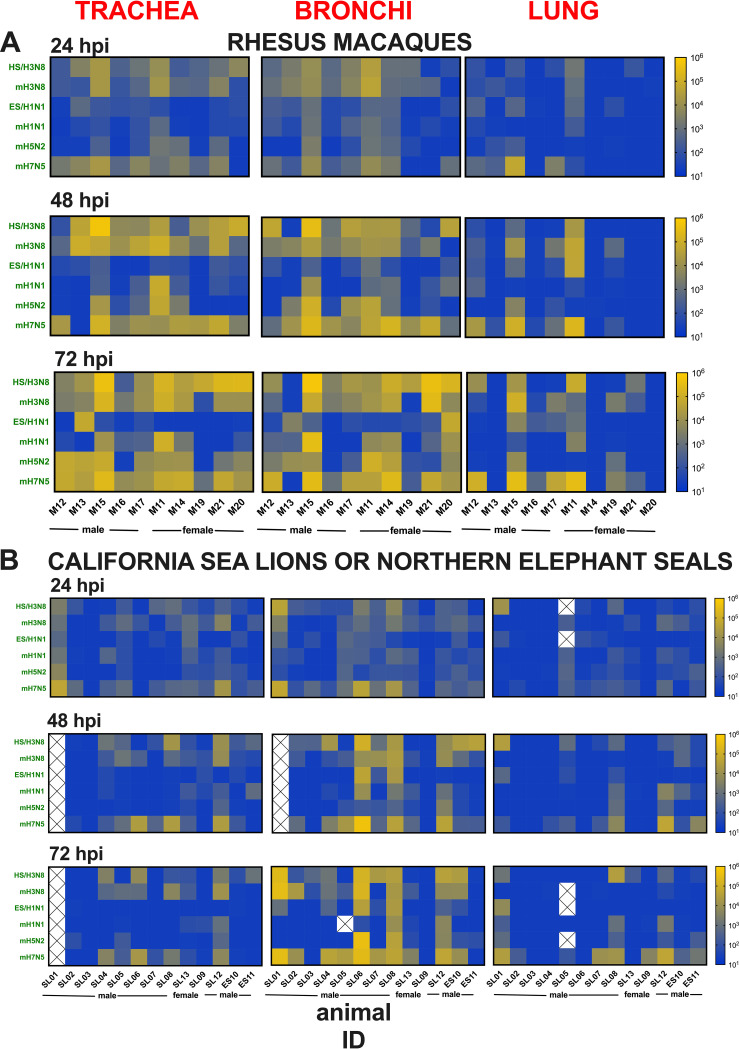
Comparison of *ex vivo* influenza A virus infection kinetics in respiratory tract explants from individual animals. (A and B) IAV titers in explants from 10 rhesus macaques (A) and 11 California sea lions and 2 Northern elephant seals (B) inoculated with 1 × 10^4^ PFU (macaques) and 2 × 10^4^ PFU (marine mammals) of each of the 6 IAV strains over a 72-h period. The kinetics of viral infection were determined by plaque assays of homogenized explants, where each square represents the titer from 10^1^ (blue) to 10^6^ (yellow) in PFU/explant. The limit of detection was 50 PFU/explant. Each explant was titrated once at 2 to 3 dilutions. Strain names refer to the original source of the virus isolate followed by the hemagglutinin and neuraminidase subtype (Table 1). HS, harbor seal. m, mallard. ES, elephant seal. Boxes with an X indicate the sample was not available for titration.

**FIG 6:**
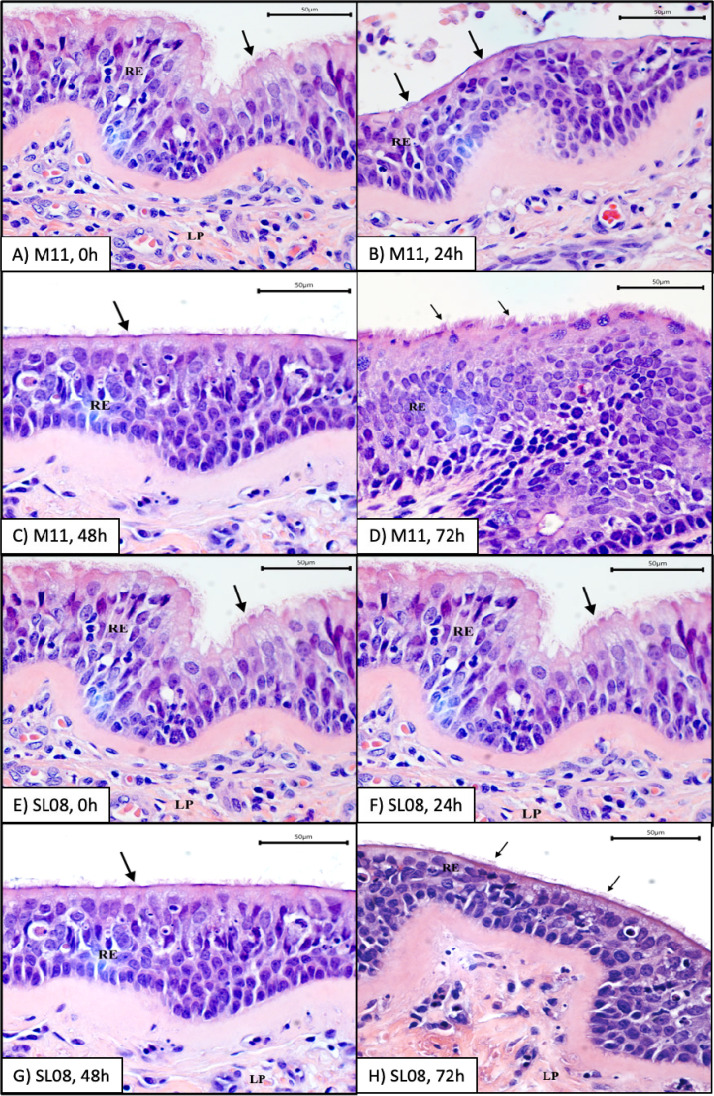
Representative light photomicrograph of sections of rhesus macaque M11 (A to D) and California sea lion SL08 (E to H) *ex vivo* tracheal explants incubated with growth medium only at 0 (A and E), 24 (B and F), 48 (C and G), and 72 h (D and H). Cilia (black arrows) are present on the apical surfaces of epithelial cells. Sections were stained with hematoxylin and eosin (×400 magnification); bar, 50 μm. RE, respiratory epithelium; LP, lamina propria.

To determine whether rhesus macaques share similar susceptibilities to different IAV subtypes, we inoculated respiratory tract explants from 10 rhesus macaques with 6 virus strains (Table 1). Five of the macaques were females and five were males, and they ranged from 1 to 16 years of age. Most of the macaques were euthanized due to injuries sustained due to nonrespiratory conditions, including trauma or chronic diarrhea. All macaques tested IAV seronegative by enzyme-linked immunosorbent assay (ELISA) in serum from blood collected at necropsy (Table 2). We focused on 0 to 72 hpi, since increases in IAV titers were observed over that period in the preliminary experiment (Fig. 2B). We first determined kinetics of mean IAV titers in explants from trachea, bronchi, and lung from all rhesus macaques considered together (Fig. 3A to C). Mean IAV infection kinetics increased from 0 to 72 hpi in all 3 explant tissues and reached highest levels at 72 hpi for most strains. For most IAV strains, slower kinetics and peak titers that were 10^2^ to 10^3^ PFU/explant lower were observed in lungs compared to trachea and bronchi. The mean area under the curve (AUC) was significantly higher for H3N8 and H7N5 strains than H1N1 and H5N2 strains in trachea and bronchi and trended higher in the lung, although not significantly (Fig. 3D). Infection kinetics in explants from a representative rhesus macaque (M15) are shown in detail (Fig. 4), and parallel strain differences were observed in explants of the means from all 10 animals. Infection kinetics in individual rhesus macaques over time (Fig. 5A) also reveal the pattern of lower titers in the lung compared to trachea or bronchi. To verify the histologic integrity of explants, hematoxylin and eosin (H&E) staining of tracheal (Fig. 6A to D) and bronchial explants (not shown) from 2 randomly selected macaques that were not IAV inoculated was performed. Microscopically, rhesus macaque tracheal and bronchial explants appeared viable for 48 to 72 h when maintained in growth medium only. Normal architecture of ciliated columnar respiratory epithelium, underlying lamina propria, submucosa, and cartilage were generally maintained. Conversely, bronchioles and alveoli were less well preserved. After 48 to 72 h in growth medium, explants exhibited variable but progressive loss of cilia at the apical epithelial surface, scattered vacuolar degeneration or single-cell necrosis of respiratory epithelial cells, and occasional small foci of epithelial cell loss, although these lesions were also occasionally observed at 0 or 24 h after incubation with medium. No explants showed evidence of cytopathic effects after 24-h incubation. Together, these results show that multiple IAV subtypes infect viable respiratory tract explants from rhesus macaques with variable kinetics and peak titers.

**TABLE 2:** Explant sources for IAV *ex vivo* infection studies^*a*^

Animal	Species	Sex	Age	Reason for euthanasia	IAV ELISA	IAV RT-PCR
M12	Macaca mulatta	Male	1 yr	Chronic diarrhea	Negative	Not performed
M13	Macaca mulatta	Male	3 yr, 4 mo	Lameness due to trauma	Negative	Not performed
M15	Macaca mulatta	Male	4 yr, 5 mo	Leg trauma	Negative	Not performed
M16	Macaca mulatta	Male	3 yr, 6 mo	Chronic diarrhea	Negative	Not performed
M17	Macaca mulatta	Male	4 yr, 5 mo	Chronic diarrhea	Negative	Not performed
M11	Macaca mulatta	Female	5 yr, 11 mo	Chronic diarrhea	Negative	Not performed
M14	Macaca mulatta	Female	16 yr, 6 mo	Endometriosis	Negative	Not performed
M19	Macaca mulatta	Female	1 yr, 6 mo	Chronic diarrhea	Negative	Not performed
M21	Macaca mulatta	Female	4 yr	Leg trauma	Negative	Not performed
M20	Macaca mulatta	Female	3 yr, 11 mo	Liver amyloid, diarrhea, bloating	Negative	Not performed
SL01	*Zalophus californianus*	Male	Yearling	Malnourished, peritonitis	Negative	Negative
SL02	*Zalophus californianus*	Male	Yearling	Leptospirosis, mild pneumonia	Negative	Negative
SL03	*Zalophus californianus*	Male	Yearling	Leptospirosis	Negative	Negative
SL04	*Zalophus californianus*	Male	Juvenile	Leptospirosis	Negative	Negative
SL05	*Zalophus californianus*	Male	Subadult	Leptospirosis, seizures	Negative	Negative
SL06	*Zalophus californianus*	Male	Subadult	Leptospirosis	Negative	Negative
SL07	*Zalophus californianus*	Male	Subadult	Leptospirosis	Negative	Negative
SL08	*Zalophus californianus*	Male	Yearling	Asphyxia	Negative	Negative
SL09	*Zalophus californianus*	Female	Adult	Domoic acid toxicity	Negative	Negative
SL12	*Zalophus californianus*	Female	Adult	Septicemia	Negative	Negative
SL13	*Zalophus californianus*	Male	Yearling	Domoic acid toxicity	Negative	Negative
ES10	*Mirounga angustirostris*	Male	Yearling	Tachypnea, tachycardia	Negative	Negative
ES11	*Mirounga angustirostris*	Male	Weanling	Blindness, congenital defect	Negative	Negative

a Tissues were derived from 10 rhesus macaques (Macaca mulatta), 11 California sea lions (*Zalophus californianus*), and 2 northern elephant seals (*Mirounga angustirostris*). M is macaque, SL is sea lion, and ES is elephant seal.

### (ii) Marine mammals.

**FIG 7:**
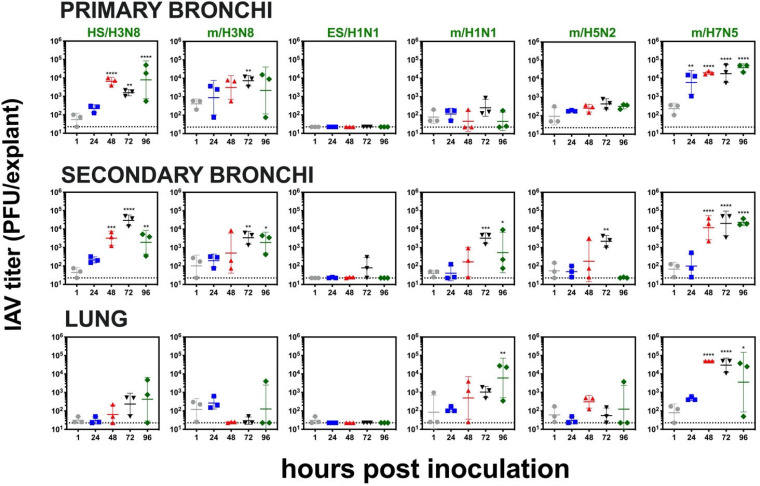
Influenza A virus infection kinetics from 1 to 96 hpi in respiratory tract explants from an adult female California sea lion (SL12 in Table 2) inoculated with 2 × 10^4^ PFU of 6 IAV strains. Viral titers are represented as the geometric mean and geometric standard deviation. Three explants at each time point were titrated independently, and the mean from the triplicates is represented by the middle horizontal line. Asterisks show *P* values (two-way ANOVA with Dunnett’s multiple comparisons) comparing titers at 24, 48, 72, or 96 hpi to 1 hpi. **, *P* < 0.005; ***, *P* < 0.001; ****, *P* < 0.0001. The dashed line indicates limit of detection, 50 PFU/explant. Strain names refer to the original source of the virus isolate followed by the hemagglutinin and neuraminidase subtype. HS, harbor seal. m, mallard. ES, elephant seal.

We next examined whether respiratory tract explants from marine mammals are susceptible to infection with the same IAV strains used to infect rhesus macaques, including the 2 strains of marine mammal origin. Explant tissues were obtained from wild California sea lions (*N* = 11) and Northern elephant seals (*N* = 2) euthanized due to nonrespiratory medical conditions at The Marine Mammal Center in Sausalito, CA. Marine mammals were mostly male and ranged in age from weanling to adult (Table 2). All animals were recovered after stranding on beaches. The causes of death varied and were sometimes ascribed to an infectious or toxic etiology but were not typically due to respiratory disease. Tissues from animals with gross respiratory tract pathologies at necropsy were also excluded from this study. All California sea lions had a mild burden of a lung worm, *Parafilaroides decorus*, which is found as a normal occurrence in healthy pinnipeds (32). Explants were sectioned to avoid worms, and no worms were observed grossly during the IAV infection studies. All marine mammals tested IAV seronegative by ELISA and IAV RNA negative by reverse transcription-PCR (RT-PCR) in nasal and rectal swabs at intake to The Marine Mammal Center (Table 2). Although tissues were used within 24 h postmortem, we confirmed ciliary viability by microscopically visualizing movement of polystyrene beads floating atop cultured California sea lion tracheal tissue from 0 to 72 h. Viability was evidenced by clearance of beads to the side of explants within 60 min. As in rhesus macaques, IAV infection kinetics for most strains increased in California sea lions from 0 to 72 hpi in all 3 tissues and reached highest levels at 72 hpi for most strains (Fig. 3E to G). Similar to rhesus macaques, the mean area under the curve (AUC) was significantly higher for H3N8 and H7N5 strains than H1N1 and H5N2 strains in trachea and bronchi, while the AUC in lung explants from sea lions was only higher for H7N5 (Fig. 3H). Detailed data (Fig. 7) from 1 representative California sea lion (SL12) paralleled patterns from the mean from all 11 animals. Infection kinetics in individual marine mammals over time (Fig. 5B) showed higher titers in the bronchi relative to trachea or lung. Contrary to our expectation that IAV of marine mammal origin would produce high titers in marine mammal explants, the strain from an elephant seal (ES/H1N1) produced lower and slower kinetics (Fig. 3E to G and 5B) than many of the strains isolated from mallards. Like rhesus macaque explants, California sea lion tracheobronchial explants inoculated with growth medium only maintained a relatively normal microscopic appearance for 48 to 72 h (Fig. 6E to H), with poor preservation of bronchioles and alveoli and progressive loss of normal architecture over time. All California sea lion tissues also exhibited variable degrees of preexisting tracheobronchitis, likely associated with their nematode burden. Although California sea lions with respiratory conditions or gross respiratory pathologies were excluded from this study, we also analyzed whether systemic conditions, including domoic acid toxicity or septicemia, may have exacerbated IAV susceptibility. We assessed AUC for IAV infection kinetics in sea lions euthanized for nonsystemic and nonrespiratory conditions, including malnourishment or leptospirosis (*N* = 6; SL01 and SL03 to -07) versus systemic conditions, including domoic acid toxicity or septicemia (*N* = 5; SL02 and SL08 to -13) (Table 2). The AUC infection kinetics between the 2 groups of animals were not significantly different in any of the three respiratory tissues (data not shown). These analyses suggest that IAV infection kinetics in wild California sea lion explants are not impacted by systemic conditions. Together, these results show that, similar to rhesus macaques, multiple IAV subtypes infect viable respiratory tract explants from marine mammals with variable kinetics and peak titers. To further determine whether the differences in mean IAV kinetics were supported statistically over time, we examined IAV infection dynamics in 24-h windows.

### (iii) IAV kinetics over time.

We tested whether explants inoculated with different IAV strains experience different mean changes in titer from 1 to 72 hpi. Considering all 3 tissue types together, the log_10_ change in viral titer was calculated for three time frames: 1 versus 24, 1 versus 48, and 1 versus 72 hpi for rhesus macaques and California sea lions (Table 3). Northern elephant seals were not included in analyses, since explants from only two animals were available, although they produced infectious IAV after inoculation with some strains (Fig. 5B). Infection kinetics of the six IAV strains differed for each time frame considered. In rhesus macaques, mean titer changes for HS/H3N8, m/H3N8, and m/H7N5 did not differ significantly from each other over any of the time frames. m/H1N1 and mH5/N2 did not differ significantly from each other over any of the time frames but were different from HS/H3N8, m/H3N8, and m/H7N5. In contrast, the change in ES/H1N1 viral titers significantly differed from all other strains except m/H1N1 from 1 to 48 hpi. In California sea lions, HS/H3N8 and m/H7N5 shared similar mean changes in log_10_ titers in all time frames. For m/H1N1 and HS/H3N8, the mean change in log_10_ titers did not vary significantly from 1 to 24 hpi but did vary from 1 to 48 and 1 to 72 hpi. The mean change in log_10_ viral titers between m/H1N1 and m/H7N5 were only different from 1 to 48 hpi and m/H5N2 and m/H7N5 differed from 1 to 48 and 1 to 72 hpi. These results show that mean changes in IAV titers over 24-h windows in respiratory tract explants from both rhesus macaques and California sea lions vary with IAV strain.

**TABLE 3:** Mean change in log_10_ titer of influenza virus strains after inoculation in rhesus macaque or California sea lion respiratory tract explant tissues^*a*^

Strain	Rhesus macaques			California sea lions		
	1 vs 24 hpi*	1 vs 48 hpi†	1 vs 72 hpi‡	1 vs 24 hpi*	1 vs 48 hpi†	1 vs 72 hpi‡
HS/H3N8	1.2 (1.1)^a^	1.8 (1.5)^ab^	2.2 (1.5)^ad^	0.4 (0.8)^e^	0.6 (1.2)^e^	0.8 (1.4)^e^
m/H3N8	0.9 (1.1)^ac^	1.8 (1.1)^ac^	0.9 (1.4)^ab^	−0.04 (0.7)^abc^	−0.07 (1.1)^abc^	0.2 (1.3)^abc^
ES/H1N1	−0.1 (0.6)^b^	−0.1 (0.8)^e^	0.1 (0.9)^e^	−0.1 (0.5)^ab^	−0.1 (0.8)^ab^	−0.1 (0.9)^ab^
m/H1N1	0.2 (0.7)^b^	0.3 (1.0)^de^	0.9 (1.2)^c^	0.03 (0.6)^bcde^	−0.05 (0.7)^bd^	0.2 (1.0)^bcd^
m/H5N2	0.3 (0.7)^bc^	1.0 (1.4)^bcd^	1.5 (1.4)^bcd^	−0.02 (0.5)^ad^	−0.07 (0.6)^ad^	0.2 (1.0)^ad^
m/H7N5	1.1 (0.8)^a^	1.9 (1.0)^a^	2.4 (1.2)^a^	0.3 (0.9)^cde^	0.5 (1.2)^ce^	0.8 (1.5)^ce^

a Means for the same time frame followed by a common letter are not significantly different by the Games-Howell test at the 5% level of significance. Values in parentheses show standard deviations. The symbols (*,†, and ‡) indicate that the mean change in titer differs significantly for at least one strain according to Welch’s ANOVA at 5% level of significance. *, 1 versus 24 hpi, F = 15.417, df = (5, 91.649), *P* = 5.55e−11 for rhesus macaques and F = 4.666, df = (5, 189.21), *P* = 4.85e−04 for California sea lions. †, 1 versus 48 hpi, F = 25.823, df = (5, 91.569), *P* = 3.59e−16 for rhesus macaques and F = 5.784, df = (5, 182.59), *P* = 5.55e−05 for California sea lions. ‡, 1 vs 72 hpi, F = 23.351, df = (5, 91.826), *P* = 4.58e−15 for rhesus macaques and F = 6.636, df = (5, 189.35), *P* = 1.02e−05 for California sea lions. Strain names refer to the original source of the virus isolate followed by the hemagglutinin and neuraminidase subtype. HS is harbor seal. m is mallard. ES is elephant seal.

### (iv) IAV kinetics by tissue.

To examine whether tissue type was associated with IAV titer, we analyzed all 6 IAV strains together (Table 4). Infection kinetics in bronchi and trachea from rhesus macaques were significantly higher than those in the lung over all 24-h windows from 1 to 72 hpi. In California sea lions, infection kinetics in bronchi and trachea were significantly higher than in the lung at 24 hpi, while infection kinetics in bronchi were significantly higher than those in the trachea and lung at 48 and 72 hpi. Together, these data support tissue-specific IAV infectivity, where bronchi and trachea from both species support production of higher IAV titers than lung explants.

**TABLE 4:** Mean change in log_10_ titer of influenza virus in respiratory tract tissues from rhesus macaques and California sea lions^*a*^

Tissue	Rhesus macaques			California sea lions		
	1 vs 24 hpi*	1 vs 48 hpi†	1 vs 72 hpi‡	1 vs 24 hpi*	1 vs 48 hpi†	1 vs 72 hpi‡
Bronchi	1.0 (0.9)^a^	1.5 (1.4)^a^	1.7 (1.5)^a^	0.3 (0.8)^a^	0.5 (1.3)^a^	0.8 (1.5)^a^
Trachea	0.8 (1.0)^a^	1.4 (1.5)^a^	1.9 (1.5)^a^	0.1 (0.7)^a^	0.1 (0.8)^b^	0.2 (1.0)^b^
Lung	0.1 (0.7)^b^	0.5 (1.1)^b^	1.0 (1.4)^b^	−0.2 (0.6)^b^	−0.1 (0.8)^b^	0.1 (1.1)^b^

a Means for the same time frame followed by a common letter are not significantly different by the Games-Howell test at the 5% level of significance. Values in parentheses show standard deviations. The symbols (*,†, and ‡) indicate that the mean change in titer differs significantly for at least one tissue according to Welch’s ANOVA at 5% level of significance. *, 1 versus 24 hpi, F = 28.071, df = (2, 127.39), *P* = 7.93e−11 in rhesus macaques and F = 15.369, df = (2, 269.45), *P* = 5.96e−05 for California sea lions. †, 1 versus 48 hpi, F = 13.641, df = (2, 129.47), *P* = 4.02e−6 in rhesus macaques and F = 10.105, df = (2, 257.71), *P* = 5.96e−05 in California sea lions. ‡, 1 versus 72 hpi, F = 7.8203, df = (2, 133.05), *P* = 6.16e−4 for rhesus macaques and F = 11.083, df = (2, 268.89), *P* = 2.37e−0.5 for California sea lions.

### (v) IAV kinetics by species.

To evaluate whether species associates with infection kinetics for the 6 IAV strains, we compared changes in mean log_10_ titer between rhesus macaque and California sea lion explants (Table 5). The mean change in titer of both H3N8 strains and m/H7N5 was significantly higher in trachea at all time points and in bronchi to 48 hpi in rhesus macaques compared to California sea lions. Changes in mean log_10_ titers for m/H3N8 in the lung from 1 to 48 and 1 to 72 hpi were higher in rhesus macaques than California sea lions. Together, these data show that rhesus macaque explants produce higher titers than California sea lions for most IAV strains used here.

**TABLE 5:** Difference in mean log_10_ change for respiratory tract explant tissues from California sea lions versus rhesus macaques^*a*^

Time frame (hpi)	IAV strain	Trachea	Bronchi	Lung
1–24	HS/H3N8	**−1.26 (0.011)**	**−1.13 (0.023)**	−0.11 (0.989)
	m/H3N8	**−1.26 (0.003)**	**−1.40 (**<**0.001)**	−0.29 (0.695)
	ES/H1N1	−0.03 (1.00)	−0.13 (0.967)	−0.10 (0.996)
	m/H1N1	−0.14 (0.959)	−0.32 (0.475)	−0.03 (1.00)
	m/H5N2	−0.41 (0.362)	−0.45 (0.388)	−0.07 (0.997)
	m/H7N5	**−1.02 (0.047)**	−0.62 (0.266)	−0.46 (0.551)
1–48	HS/H3N8	**−1.95 (0.002)**	−1.27 (0.120)	−0.18 (0.982)
	m/H3N8	**−2.21 (<0.001)**	**−1.50 (0.009)**	**−1.50 (0.006)**
	ES/H1N1	−0.02 (1.00)	0.25 (0.963)	−0.45 (0.572)
	m/H1N1	−0.74 (0.160)	−0.48 (0.714)	0.01 (1.00)
	m/H5N2	−0.99 (0.100)	**−1.50 (0.024)**	−0.25 (0.918)
	m/H7N5	**−1.75 (<0.001)**	**−1.50 (0.001)**	−0.71 (0.540)
1–72	HS/H3N8	**−2.41 (<0.001)**	**−1.74 (0.015)**	−0.49 (0.711)
	m/H3N8	**−2.02 (<0.001)**	−1.44 (0.085)	**−1.87 (0.006)**
	ES/H1N1	−0.05 (1.00)	0.01 (1.00)	−0.48 (0.464)
	m/H1N1	−0.83 (0.265)	−1.06 (0.131)	−0.03 (1.00)
	m/H5N2	**−2.11 (<0.001)**	−1.23 (0.083)	−0.32 (0.893)
	m/H7N5	**−2.16 (<0.001)**	−1.08 (0.229)	−1.11 (0.335)

a Differences are shown as *P* values after the Games-Howell *post hoc* test. Significant *P* values (*P < *0.05) are in boldface. Strain names refer to the original source of the virus isolate followed by the hemagglutinin and neuraminidase subtype. HS is harbor seal. m is mallard. ES is elephant seal.

### Cellular tropism of influenza A virus in rhesus macaque and California sea lion explants.

**FIG 8:**
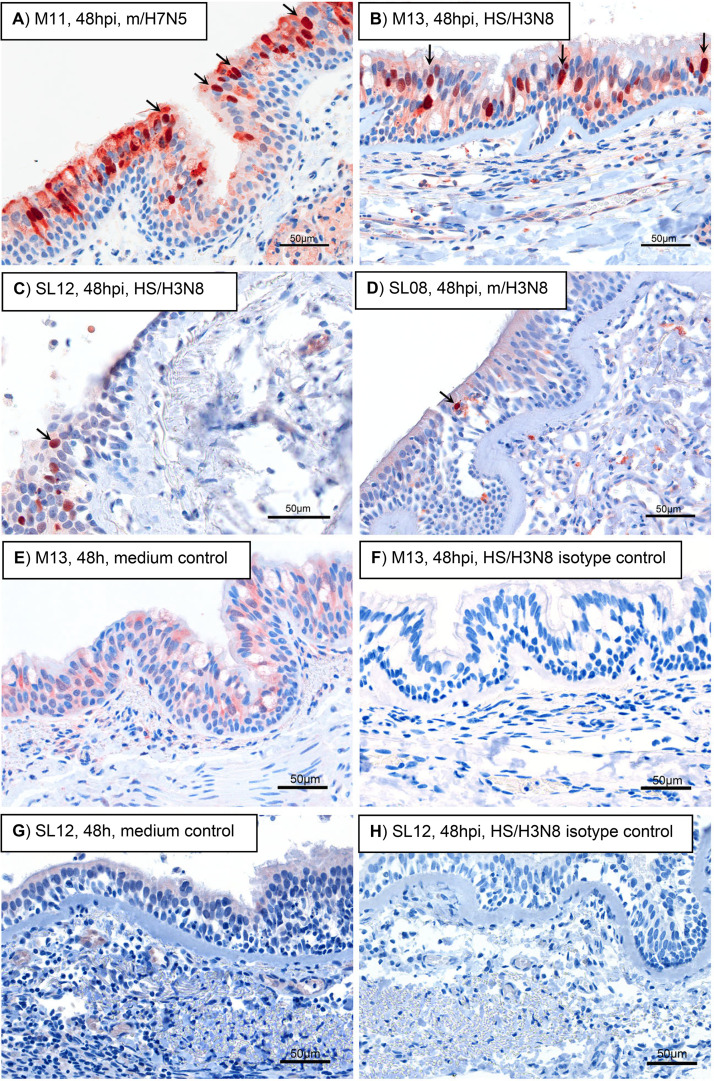
Immunohistochemical (IHC) staining for influenza A virus in rhesus macaque and California sea lion tracheal and bronchial explants. (A and B) Bronchi from rhesus macaques 48 h postinoculation (hpi) with m/H7N5 (animal M11) (A) or HS/H3N8 (animal M13) (B). (C) Trachea from California sea lion SL12 48 hpi with HS/H3N8. (D) Trachea from California sea lion SL08 24 hpi with m/H3N8. (E and F) Bronchi from rhesus macaque M13, 48 hpi, treated with growth medium only (E) or with HS/H3N8 (F) and stained with isotype IgG. (G and H) Trachea from SL12 48 hpi, treated with growth medium only (G) or with HS/H3N8 (H) and stained with isotype IgG. IHC stain used an antibody that labels IAV nucleoprotein. Positive respiratory epithelial cells exhibit strong nuclear immunoreactivity (red-brown nuclear staining; arrows). Positive staining is primarily localized to apical epithelial cells, with relative sparing of basal cells.

We used immunohistochemistry to assess the cellular tropism of IAV in rhesus macaque and California sea lion respiratory tissue explants (Fig. 8, Table 6). At least 22 sections from each tissue type for 2 rhesus macaques (M11 and M13) and at least 56 sections from each tissue type for 2 California sea lions (SL08 and SL12) were evaluated. All examined sections exhibited variable degrees of nonspecific cytoplasmic, membranous, and/or background staining; as such, only cells with strong nuclear immunoreactivity were considered positive. For all explants from both species and all IAV subtypes, positive staining for viral nucleoprotein was limited to epithelial cells of the trachea and bronchi; representative slides are shown (Fig. 8A to D). Staining for IAV nucleoprotein was not observed in non-inoculated explants or IHC antibody-treated negative isotype controls from rhesus macaque (Fig. 8E and F) or California sea lion (Fig. 8G to H) trachea. Pneumocytes within the lung were IAV negative. Infection was primarily localized to apical epithelial cells, particularly tracheal and bronchial ciliated columnar cells and goblet cells, while basal cells were largely spared. Rhesus macaque explants from M11 and M13 infected with H3N8 strains exhibited higher relative proportions of positive tracheal and bronchial epithelial cells 24 and 48 hpi, even though IAV titers at those times for most strains exceeded 10^4^ PFU/explant 24 hpi (Fig. 5A). The explants from M11 infected with m/H7N5 exhibited the largest variation in relative proportion of positive cells between 24 and 48 hpi. The number of infected respiratory epithelial cells generally increased between 24 and 48 hpi, which is consistent with infection kinetics where IAV titers increased during that period. While positive nuclei were less frequent within California sea lion explants, those infected with H3N8 strains also exhibited higher relative proportions of positive tracheal and bronchial epithelial cells at 24 and 48 hpi, which is consistent with data from rhesus macaques. These results demonstrate that IAV infects the apical epithelial cells of upper respiratory tract explants in rhesus macaques and California sea lions.

**TABLE 6:** Immunohistochemical detection of influenza A virus in macaque and California sea lion respiratory tissue explants^*a*^

Animal	No. of IAV-positive epithelial cells/mm^2^ by hpi							
	M11		M13		SL08		SL12	
	24	48	24	48	24	48	24	48
HS/H3N8	458	519	71	224	0	0	51	21
m/H3N8	349	544	0	1	2	0	0	26
ES/H1N1	0	0	0	0	0	0	0	0
m/H1N1	0	8	0	0	0	0	0	0
m/H5N2	7	19	0	0	0	0	0	0
m/H7N5	50	626	9	0	0	0	8	<5

a Trachea, bronchi, and lung were examined for each animal. At least 22 sections from each tissue type were examined for the rhesus macaques, and at least 56 sections from each tissue type were examined for the California sea lions. Strain names refer to the original source of the virus isolate followed by the hemagglutinin and neuraminidase subtype. HS is harbor seal. m is mallard. ES is elephant seal.

### Genetic changes in influenza A viruses sequenced from explants.

IAV genomes in inoculated explants from 2 rhesus macaques, 3 California sea lions, and 1 Northern elephant seal were compared to sequences of the strains used as inocula (Table 7). Sequence comparisons showed that all genomes from rhesus macaque explants were 100% identical at the consensus level (mutations occurring on 50% or more of viral RNAs) to their corresponding inoculum (data not shown). Six nonsynonymous nucleotide substitutions in the HA, NA, and PA genes were detected in California sea lion and Northern elephant seal explants inoculated with different IAV subtypes (Table 8). Inocula contained a mixture of bases including the mutant nucleotide at each of these loci, indicating that none of the mutations developed *de novo* in explants (data not shown). One synonymous nucleotide substitution, PB2 1368 in m/H7N5, was detected in bronchi from all 3 California sea lions at 48 and 72 hpi. The nucleotide affecting this substitution was present at a minority frequency (41%) in the inoculum, indicating that it did not develop *de novo* but increased to consensus frequency in explants by 48 hpi (data not shown). Together, these data show that no *de novo* IAV mutations were detected in any strain during *ex vivo* respiratory tract explant infection from selected rhesus macaques and marine mammals.

**TABLE 7:** IAV-inoculated rhesus macaque and marine mammal respiratory tract explants from which whole-genome IAV sequences were obtained at indicated times postinoculation and compared to their respective inocula^*a*^

Animal ID	Tissue	hpi					
		HS/H3N8	m/H3N8	ES/H1N1	m/H1N1	m/H5N2	m/H7N5
M15	Trachea	72	72		72	72	72
	Lung						72
M11	Trachea	72	72		72		72
SL01	Bronchi		72				72, 96
SL06	Bronchi	48, 72	72			72	48, 72
SL08	Trachea						48
	Bronchi			72			72
ES10	Bronchi	48, 72					

a Empty spaces indicate samples for which sequencing was not attempted. ES is elephant seal. M is macaque. SL is California sea lion.

**TABLE 8:** IAV amino acid changes compared to inocula detected in *ex vivo* bronchi explants from California sea lions and a Northern elephant seal^*a*^

Animal ID	hpi	HS/H3N8 HA	HS/H3N8 NA	ES/H1N1 PA	m/H7N5 HA	m/H7N5 PB2^*b*^
SL01	72	X	X	X	V311I	1368
	96		V394I	X		
SL06	48			X		1368
	72		P118S	X		1368
SL08	48	X	X	X		1368
	72	X	X	I359N, M441I		1368
ES10	48	A154T	P118S	X	X	X
	72	A154T	P118S	X	X	X

a Numbers shown correspond to positions in indicated IAV proteins. Empty spaces indicate no sequence differences were detected. An X shows samples for which sequencing was not attempted. HS is harbor seal, m is mallard, ES is elephant seal, M is macaque, SL is California sea lion, HA is hemagglutinin, NA is neuraminidase, PA is polymerase acidic protein, and PB is polymerase basic protein.

b The mutation was a synonymous change.

## DISCUSSION

We adapted an *ex vivo* respiratory tract infection model to study susceptibility and to define comparative infection kinetics of IAV in colony-raised rhesus macaques and wild marine mammals. We observed that IAV exhibits temporal, subtype- and species-dependent infection kinetics in explants from both rhesus macaques and California sea lions. Although the relative infection kinetics for the six strains was similar for explants from rhesus macaques and California sea lions, similar patterns were not observed in MDCK cells. This suggests that immortalized cell lines do not accurately represent infection phenotypes *in vivo*, further underscoring the value of using *ex vivo* systems.

*Ex vivo* respiratory tract models have been used for studying respiratory pathogens of humans, swine, bovines, canines, and equines (23–28, 33–35). *Ex vivo* cultures of respiratory tissues provide a close resemblance to the respiratory tract *in vivo* by (i) shared polarity, where the basolateral surface is exposed to culture medium and the apical cell surface is exposed to air, (ii) shared cell positioning *in situ*, which maintains virus receptor distribution, (iii) possession of multiple cell types and states of differentiation, (iv) maintenance of the three-dimensional integrity and architecture of a tissue, unlike cell monocultures, and (v) ciliary activity to preserve mucociliary clearance. Given these similarities to living IAV hosts, explants are valuable tools for assessing host susceptibility, infection kinetics, and pathogenesis of respiratory pathogens. *Ex vivo* systems also better assess infectivity than cell monoculture binding assays that only measure virus-receptor affinity (25, 27). In addition, tissues collected from a single animal can be divided into pieces and used to compare relative infectivity of different viral strains while holding the host constant. Lastly, use of explants from animals euthanized for other reasons reduced the number of animals used in research, following the principles of reduction, replacement, and refinement.

Limitations of the *ex vivo* approach include restricted availability of tissues, short periods of viability, and interanimal variability in IAV susceptibility. The absence of a blood supply also constitutes a weakness, since recruitment of immune cells to the infection site cannot occur. However, such a pitfall might be coopted in future studies to understand the effects of innate and intrinsic immunity without immune cell influx. Use of explants from wild marine mammals euthanized for nonrespiratory conditions, some of which present systemic conditions, is another limitation of this study. Some of the marine mammals used in this study showed evidence of exposure to toxins, malnourishment, or congenital defects, factors that may modify IAV susceptibility. However, systemic conditions, including toxin exposure, did not correspond to observed differences in IAV infection capacity in California sea lion explants in the current study. Given that the only marine mammals available for this study were those euthanized for other conditions, we unfortunately do not have the opportunity to study tissues from healthy animals, especially since both California sea lions and elephant seals are protected by the Marine Mammal Protection Act.

Lower IAV titers in *ex vivo* rhesus macaque and marine mammal lungs relative to trachea and bronchi support the upper respiratory tract as the primary site of virus infection with the virus strains used in this study. The ability of IAV to produce higher titers in the upper respiratory tract may reflect adaptation of the virus to infect cell targets in closest anatomic proximity to its entry point and shedding site, the respiratory mucosa. Lower susceptibility of the lung to IAV infection could result from decreased receptor expression in that tissue. We did not identify or enumerate IAV receptor expression in explants in this study to confirm this hypothesis. An alternate possibility is that lungs of both species decayed more quickly than the trachea and bronchi, rendering them less susceptible to IAV infection. However, we feel accelerated lung decay is unlikely, given our gross and histologic observations showed similar architectural integrity in all 3 tissues to 72 hpi (data not shown). Alternatively, lower IAV lung infectivity could result from increased resident immune cell activation and response in that tissue.

The infection kinetics of harbor seal-origin H3N8 and mallard-origin H7N5 in California sea lion respiratory tract explant tissues were superior to those of other subtype strains isolated from mallard- and elephant seal-origin H1N1. Since all strains share similar passage histories, infection kinetic differences likely are not caused by varying passage or changes in consensus sequence, since all were nearly identical to those of their unpassaged progenitors. Our observation that the elephant seal H1N1 infection kinetics were lower than those of other strains in California sea lions suggests that having a marine mammal as the isolate source does not necessarily translate to higher marine mammal explant infectivity. The lack of augmented infectivity in explants of the species from which the isolate was made has also been observed in swine, where strains of the same subtype isolated from both swine and humans showed similar infection kinetics (36). Further, some human-isolated IAV show great interstrain variability in infection kinetics in human explants (27). Despite the different infection kinetics across strains, the 6 strains used here, representing 4 IAV subtypes, productively infected at least one explant type for both species. These data suggest that any of these subtypes also infect marine mammals in the wild, consistent with serologic data showing exposure to many subtypes (13–16, 18, 19). Together, data presented here show that the marine mammal *ex vivo* culture of respiratory tissues is a tool to study IAV susceptibility, host range, and tissue tropism.

## MATERIALS AND METHODS

### Ethics statement.

This project was conducted with approval from the United States National Marine Fisheries Service Marine Mammal permit number 18786-04. The University of California, Davis, is accredited by the Association for Assessment and Accreditation Laboratory Animal Care International (AAALAC). Animal care was performed in compliance with the 2011 *Guide for the Care and Use of Laboratory Animals* provided by the Institute for Laboratory Animal Research (37). Rhesus macaque studies were approved by the University of California, Davis, IACUC under protocol number 19760.

### Cell culture.

Madin-Darby canine kidney (MDCK) cells (ATCC CCL-34) were maintained at 37°C and 5% CO_2_ in MDCK growth medium (Iscove's modified Dulbecco's medium [IMDM] supplemented with 5% fetal bovine serum [FBS], 0.1% sodium bicarbonate, penicillin, and streptomycin).

### Influenza A virus propagation and titration.

Since marine mammals and water birds share the same near-shore environments where cross-species transmission can occur, we used strains isolated from both marine mammals and birds (Table 1). The strains used also represent common IAV subtypes. Further, the elephant seal (ES)/H1N1 strain shows high genetic similarity with human pandemic H1N1 (4). Strains were isolated from an elephant seal (*N* = 1), a harbor seal (*N* = 1), or mallard ducks (*N* = 4), and they share similar passage histories in embryonated chicken eggs and MDCK cells. The harbor seal H3N8 (HS/H3N8) IAV strain was obtained from Hon Ip (National Wildlife Health Center, Madison, Wisconsin). The other strains were isolated at UC Davis and used in this study. Avian allantoic fluid virus stocks of 6 strains were amplified in MDCK cells to obtain sufficient titers and volumes for use as inocula. Viral titers of the 6 inocula were determined by plaque assay using MDCK cells. Briefly, MDCK cells were grown in 6-well plates to 80 to 90% confluence in MDCK growth medium. Viral stocks were serially 10-fold diluted in viral growth medium (minimum essential medium [MEM] supplemented with 0.5% bovine serum albumin [BSA], 0.1% sodium bicarbonate, 10 mM HEPES, penicillin, and streptomycin). The MDCK cells were washed 3 times with Dulbecco’s phosphate-buffered saline (DPBS) prior to inoculation with 200 μl of diluted virus samples. After 1 h of incubation at 37°C with 5% CO_2_, 3 ml viral growth medium containing 1 μg/ml *N*-*p*-tosyl-l-phenylalaninechloromethyl ketone (TPCK) and 0.5% agarose was added to each well. After a 48-h incubation at 37°C with 5% CO_2_, MDCK cells were fixed with 4% formaldehyde and stained with 0.05% crystal violet.

### *In vitro* infection kinetics of IAV.

We compared relative infection kinetics for the 6 IAV strains used in this study. Infection kinetics for the 6 IAV strains were assessed in MDCK cells. Cells were grown at 37°C with 5% CO_2_ to 6 × 10^5^ cells/well in 24-well plates and inoculated in triplicate with 6 × 10^3^ PFU virus of IAV, representing a multiplicity of infection (MOI) of 0.01. After 1 h of incubation, each well was washed with 1 ml of DPBS 3 times to remove unbound virus. Next, viral growth medium supplemented with 1 μg/ml TPCK trypsin for IAV was added to each well. The supernatant was sampled at 1, 24, 48, and 72 h postinoculation and stored at −80°C in virus growth medium. Viral titers were assessed by plaque assay.

### Titration to quantify infectious IAV.

Plaques were counted against a white background, and the titer (in PFU) was determined by counting wells with individual plaques. Two to 3 dilutions of each explant were tested once. Viral titers were recorded as the reciprocal of the highest dilution where plaques are noted and represented as number of PFU per milliliter for liquid samples or number of PFU per explant for tissues. The limit of detection (LOD) of the plaque assay was 50 PFU/ml or explant.

### Explant sources, collection, and processing.

Respiratory tract tissues, including trachea, bronchi, and lungs, were used for explant studies (Table 2). Tissues were obtained from Indian-origin rhesus macaques (Macaca mulatta), euthanized due to medical conditions, that were born and raised at the California National Primate Research Center, University of California, Davis, CA. The respiratory tract tissues of marine mammals were from wild California sea lions (*Zalophus californianus*) and Northern elephant seals (*Mirounga angustirostris*) that were stranded on beaches and were euthanized due to medical conditions at The Marine Mammal Center in Sausalito, CA. The Marine Mammal Center rehabilitates stranded marine mammals. Some animals that present with severe conditions are not able to be returned to the wild and are therefore euthanized. To reduce confounding effects of respiratory disease on IAV infection, we intentionally excluded animals whose necropsy reports indicated respiratory disease as the primary or sole reason for euthanasia. Sera harvested from blood collected at necropsy from macaques and marine mammals were tested for IAV antibody directed against a conserved epitope of the nucleoprotein using an enzyme-linked immunosorbent assay (ELISA), ID-Screen influenza A antibody competition multispecies kit (IDvet, Grabels, France) by following the manufacturer’s instructions. Positive and negative controls included in the kit were also tested on each plate. An ELX808 BioTek spectrophotometer (BioTek Instruments, Winooski, VT) was used to measure absorbance. Serum was considered positive for IAV antibody when the ratio of the absorbance of the test sample to the negative control was less than 0.45, as previously established (21, 22). Marine mammals were also tested for IAV RNA by reverse transcription PCR (RT-PCR). Nasal and rectal swabs were collected from each California sea lion and Northern elephant seal by veterinary staff at The Marine Mammal Center upon entry to the facility. Swabs were placed in vials containing 1.5 ml of viral transport medium (VTM). Samples were refrigerated for up to 1 week prior to shipping to the laboratory. Once received at the laboratory, swab samples were processed the same day or stored at −80°C. RNA was extracted from swab samples using the MagMAX-96 AI/ND viral RNA isolation kit (Applied Biosystems, Foster City, CA) and a KingFisher magnetic particle processor (Thermo Scientific, Waltham, MA). Extracted RNAs from swab samples were subjected to an established IAV RT-PCR (38) targeting a conserved region of the matrix gene using the AgPath-ID one-step RT-PCR mix (Applied Biosystems, Foster City, CA) and an ABI 7500 real-time PCR system (Applied Biosystems, Foster City, CA). The positive control, a cell culture isolate of ES/H1N1, and a negative control, VTM, were also tested on each plate. Samples with a cycle threshold (*C_T_*) value of <45 were considered positive (39). Tissues from all three species were stored for up to 24 h in Roswell Park Memorial Institute 1640 (RPMI) medium (Gibco) at 4°C. During tissue preparations, lungworms (*Parafilaroides decorus*) were observed, either in airways (i.e., bronchioles/bronchi) or sometimes free in the lung parenchyma (in which case they were occasionally associated with mild inflammation) in all California sea lions. Areas of tissues without worms were selected as explants. No worms were observed grossly in explants during the IAV infection studies. Prior to preparation for infection studies, tissues were separated and washed 4 to 6 times in 50-ml conical tubes containing 30 ml of RPMI medium for 2 to 5 min each at 22°C. The final wash was performed in RPMI growth medium (RPMI medium supplemented with 100 U/ml each of penicillin and 100 μg/ml streptomycin [Gibco] and 1× antibiotic-antimycotic [Gibco]).

### Preparation of tracheal, bronchi, and lung explants.

A simplified *ex vivo* culture procedure was derived from the methods described by Nunes et al. (23). After washing in RPMI medium, the surrounding connective tissue exterior to the tracheal and bronchi cartilage was removed. Tracheas were cut into O-rings horizontally where each slice contained about 0.5-cm cartilage. Each O-ring consisted of the respiratory mucosa, epithelial cell layer, and underlying cartilage. O-rings were further cut into small pieces of approximately 0.5 cm by 0.5 cm. Bronchi were cut similarly. Explants were implanted singly onto agarose plugs (RPMI growth medium containing 0.5% agarose) in 24-well plates with the mucosal surface facing up. Explants were maintained in a humidified 37°C incubator with 5% CO_2_ for up to 7 days.

### *Ex vivo* IAV infection kinetics.

To evaluate IAV infection kinetics *ex vivo*, trachea, bronchi, and lung explants were inoculated in triplicate (when sufficient tissue was available) with 1 × 10^4^ PFU of one of each of the 6 IAV strains in 10 μl viral growth medium onto the epithelial surface of each section for rhesus macaques. The inoculum volume for marine mammals was adjusted to 20 μl at a dose of 2 × 10^4^ PFU for each IAV strain. Viral growth medium was used in the mock-infected control explants. Inoculated explants were sampled at 1 h postinoculation (hpi) and every 24 h up to 72 hpi (or as indicated in graphs) for viral quantification and histology. For viral quantification, each infected explant was directly immersed in 0.5 ml RPMI growth medium in a 2-ml centrifuge tube with a 5-mm glass bead and homogenized in a Mixer Mill MM300 (Retsch, Leeds, UK) at 30 Hz for 4 min at 22°C, followed by centrifugation at 16,000 × *g* for 4 min and storage at −80°C. Titers of the released progeny virus from explants were determined by plaque assay. Titers are reported as the geometric means from triplicate explants at each time point.

### Stability assays.

To determine whether IAV detected after inoculation was due to productive virus infection in respiratory tissues, we performed viral stability assays in the absence of tissue sections. Medium containing 1 × 10^4^ PFU/ml of each of the 6 IAV strains was added to 24-well tissue culture plates, and supernatant was collected at 0, 24, and 48 hpi for the determination of viral titers by plaque assay.

### IAV immunohistochemistry.

Tracheal, bronchial, and lung explants from a subset of IAV-inoculated animals (M11, M13, SL08, and SL12) were fixed in 10% buffered formalin and embedded in paraffin. Explants not treated with IAV and incubated only with rabbit IgG (Invitrogen) as the primary antibody were included as negative isotype controls for background staining. These animals were selected since they showed higher IAV titers than others. Antigen retrieval was performed on 5-μm sections via incubation in AR10 (Biogenex) in a digital decloaking chamber (Biocare Medical) for 2 min at 125°C, followed by cooling to 90°C for 10 min, rinsing with water, and a final rinse in Tris-buffered saline with 0.05% Tween 20. Tissue sections were exposed to the primary rabbit anti-influenza A virus nucleoprotein antibody (LSBio) at a ratio of 1:250 with antibody diluent (Dako). Tris-buffered saline with 0.05% Tween 20 was used for all washes. Nonspecific binding sites were blocked with 5% bovine serum albumin (Jackson ImmunoResearch). Binding of the primary antibody was detected using Envision rabbit polymer with aminoethyl carbazole as the chromogen (Dako). Each tissue section was evaluated independently by two pathologists. Sections from slides were visualized with a Zeiss Imager Z1 (Carl Zeiss). Digital images were captured and analyzed using Openlab software (Improvisation). Cells with nuclear immunoreactivity were considered positive. The positive cells in the epithelium of the trachea were manually counted. The area of the tracheal epithelium was measured. The number of positive cells was presented as cells per square millimeter of the epithelium.

### Influenza A virus genome sequencing.

IAV RNA from inocula and homogenized explant samples harvested 48 to 96 hpi from selected animals for all 3 species were sequenced. Viral RNAs were extracted using the Magmax-96 AI/ND viral RNA isolation kit (Applied Biosystems). Viral RNA extracts were used as a template for multisegment RT-PCRs to generate all 8 genomic segments of IAV using procedures described previously (22, 40). Consensus sequences were generated at the Icahn School of Medicine at Mount Sinai as described previously (41). Genome sequences from explant samples were compared to inocula.

### Statistical analyses.

Statistical analyses were conducted using GraphPad Prism software version 8. Two-way ANOVAs with Dunnett’s multiple-comparison tests were performed to compare the mean viral titers in explants at 24, 48, and 72 hpi to the mean titer 1 hpi as well as mean viral titers in MDCK cells for all 6 IAV strains at 24, 48, and 72 hpi. IAV titers in tissue explants were plotted at 0, 24, 48, and 72 hpi, and mean area under the curve (AUC) was calculated for all titers above the assay limit of detection. Within individual tissues, mean AUC was compared by one-way ANOVA using Tukey’s method for multiple comparisons between all IAV strains. AUC for California sea lions grouped by reason for euthanasia (respiratory versus nonrespiratory) were compared by Mann-Whitney rank test. Mean titers for IAV strains and tissues after inoculation of rhesus macaque and California sea lion explants were computed using R (42). Welch ANOVA with the Games-Howell *post hoc* tests were used to compare changes in mean log_10_ viral titers from 1 to 24, 48, and 72 hpi across IAV strains, tissues within species, and by IAV strain and tissue pairs between species.

### Data availability.

Sequencing data are available at the CEIRS Data Processing and Coordinating Center (DPCC) under project identifier SP4-Boyce_5004 and Submission_ID 1136547734004 and at GenBank under accession numbers MW132167–MW132399.
